# Cytological and Molecular Mechanism of Low Pollen Grain Viability in a Germplasm Line of Double Lotus

**DOI:** 10.3390/plants12020387

**Published:** 2023-01-13

**Authors:** Guangyang Liu, Fengjun Liu, Huiyan Jiang, Jun Li, Jing Jing, Qijiang Jin, Yanjie Wang, Ping Qian, Yingchun Xu

**Affiliations:** 1 Key Laboratory of Landscape Agriculture, Ministry of Agriculture and Rural Affairs, Key Laboratory of Biology of Ornamental Plants in East China, National Forestry and Grassland Administration, College of Horticulture, Nanjing Agricultural University, Nanjing 210095, China; 2 Suzhou Academy of Agricultural Sciences, Suzhou 215000, China; 3 Hangzhou West Lake Scenic Area Management Committee, Hangzhou 310013, China

**Keywords:** double lotus, self-pollination seed setting rate, tapetum, transcriptome, *NnPTC1*

## Abstract

Self-fertilization rate is an essential index of lotus reproductive system development, and pollen activity is a key factor affecting lotus seed setting rate. Based on cytology and molecular biology, this study addresses the main reasons for the low self-set rate of double lotus. It takes two different double lotus breeds into consideration, namely ‘Sijingganshan’ with a low self-crossing rate and ‘Jinfurong’ with a high self-crossing rate. Cytological analysis results showed that the pollen abortion caused by excessive degradation of tapetum during the single phase was the root cause for the low self-mating rate of double lotus. Subsequent transcriptome analysis revealed that the gene *NnPTC1* related to programmed tapetum cell death was significantly differentially expressed during the critical period of abortion, which further verified the specific expression of *NnPTC1* in anthers. It was found that the expression level of *NnPTC1* in ‘Sijingganshan’ at the mononuclear stage of its microspore development was significantly higher than that of ‘Jinfurong’ at the same stage. The overexpression of *NnPTC1* resulted in the premature degradation of the tapetum and significantly decreased seed setting rate. These results indicated that the *NnPTC1* gene regulated the pollen abortion of double lotus. The mechanism causing a low seed setting rate for double lotus was preliminarily revealed, which provided a theoretical basis for cultivating lotus varieties with both flower and seed.

## 1. Introduction

*Nelumbo nucifera Gaertn* (lotus) is a famous traditional Chinese flower with a beautiful appearance and abundant germplasm resources. Lotus seeds are rich in starch, protein, polysaccharides, amino acids, mineral elements, phenols, alkaloids and other substances [1,2] with antioxidant, anti-aging, and kidney protection, and they reduce free radicals in the body [3,4] and are an excellent health food. Double lotus is one kind of lotus with high ornamental value. However, the self-pollination and seed setting rate of most double lotus flowers is low (<50%) [5], which cannot meet the demand for lotus seed production of a single variety. As a variety that integrates the advantages of lotus and flower lotus, it can significantly meet market requirements and increase farmers’ income. However, few varieties, such as ’Jinfurong1’, are currently widely used [6]. Therefore, to solve the problems related to low fruiting of double lotus, we can improve it genetically and promote the development and application of new varieties of recognition and dual-use lotus.

Plant sexual reproduction disorder mainly focuses on male sterility, fertilization disorder and embryo abortion after fertilization. Male sterility, pollen grain malformation and pollen grain vigor decreasing are essential factors affecting seed setting rate. Pollen grain abortion caused by low pollen grain viability may occur at any stage of anther development, and there are significant differences among different species [7]. Abnormal development of microspores can happen at any growth and development stage, ranging from the spore-cell stage to the mature pollen grain stage, and may lead to pollen grain abortion [8]. By counting the occurrence period of pollen grain abortion of a large number of male sterile plants, Kempken found that the pollen grain abortion of monocotyledon plants began after the production of monocyte pollen grain. In contrast, the pollen grain abortion of the majority of dicotyledon plants was mainly concentrated from meiosis to tetrad [9]. Previous cytological studies on male sterility types of cabbage species showed that the periods of pollen grain sterilization were in the cytological stage, the prophase of tetrad, the pollen grain mother cell stage and the late phase of the tetrad. The abortion of the first three types of male sterility is related to the abnormal development of the velvet layer, and the last type is caused by the abnormal division of tetrad [10].

Tapetum plays an extremely important role in pollen development [11,12]. The abnormal development of tapetum cells leads to premature or delayed degradation of tapetum, which will affect pollen grain development, resulting in pollen grain malformation and reduced vitality [13,14,15], thus leading to male sterility. Studies have shown that tapetum can provide nutrients and structural materials for pollen grain, and it can synthesize and secrete callose enzymes to decompose the callose wall of pollen grainmother cells and tetrads, so as to release and develop single pollen grains in young stages [16,17].

Transcriptomics is a discipline that systematically studies transcriptional gene mapsat the global transcriptional level and reveals the molecular mechanisms of complex biological pathways and trait regulatory networks. Based on previous studies, transcriptome sequencing technology has been widely used on plant fertility-related genes. For example, in the study of male sterility in chrysanthemums, genes related to abortion, such as *ERF*, *AMS* and *MS*, were screened by transcriptome sequencing [18]. In the study of grape embryo abortion” seedless-related candidate genes were obtained through RNA-Seq screening. Studies have shown that HD-Zip transcription factors are closely related to grape embryo abortion, such as *VvHDZ28* and *VvHDZ27* [19]. Transcriptome analysis in maize also found that microRNA regulated S-type cytoplasmic male sterility [20].

Previous studies have revealed that some genes can regulate tapetum degradation at the later stage of pollen grain development, thus affecting grain fertility. As a transcription factor, MALE STERILITY 1 (*MS1*) with PHD domain has been identified to be involved in the degradation of anthers tapetum in both Arabidopsis barley and is considered an essential gene for pollen grain development [21]. In rice, *MS1* homolog *PTC1* also participates in pollen grain development by regulating tapetum development [22]. Plant homeodomain (PHD) is a conserved zinc-finger domain in the evolution of eukaryotes. Proteins containing the PHD domain can be used as transcription factors to participate in various life activities of plants, such as embryonic meristem germination, flowering and meiosis, and the subsequent development of pollen grain after meiosis [23]. *TIP3*, a gene containing the PHD domain in rice, acts as a transcriptional activator and interacts with the bHLH-type transcription factor TDR, thereby affecting programmed cell death in the tapete [24].

In this study, the double lotus variety ‘Sijinggangshan’ with a low self-fertilization rate and ‘Jinfurong 1’ with a high self-fertilization rate were used as research materials to reveal the main reasons for the low self-fertilization rate of double lotus and to explore the mechanism of low pollen grain vitality of double lotus variety from the perspectives of cytology, transcriptomics and molecular biology. In addition, this study proposed a new mechanism of the *NnPTC1* transcription factor for the fertility of lotus pollen grains, providing theoretical guidance for the breeding of lotus for food and appreciation.

## 2. Results

### 2.1. Self-Pollination and Embryo Development

After one month of artificial pollination, the self-breeding and fruiting of the two varieties were counted (Table 1). The selfing seed setting rate of ‘Jinfurong 1’ was 63.33%, while that of ‘Sijinggangshan’ was only 7.09%. The selfing seed setting rate of ‘Jinfurong 1’ was significantly higher than that of ‘Sijinggangshan’. Compared with the natural open pollination seed set rate, the artificial self-pollination seed set rate of the two varieties decreased significantly, and the decline range of ‘Sijinggangshan’ was greater (Table S1).

**Figure 1:**
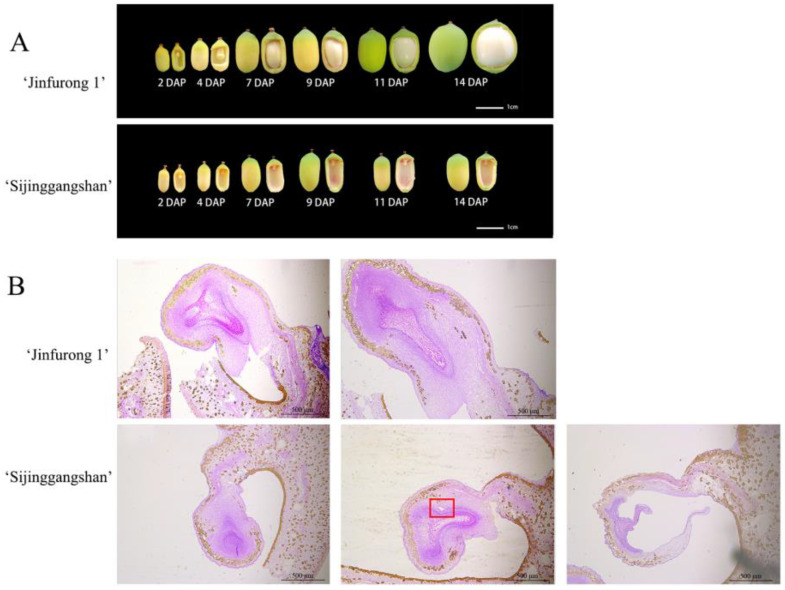
Embryo Development of ‘Jinfurong 1’ and ‘Sijinggangshan’. (**A**) Ovule development process of ‘Jinfurong 1’ and ‘Sijinggangshan’ after artificial self-pollination. (**B**) The anatomy of embryonic development of ‘Jinfurong 1’ and ‘Sijinggangshan’ DAP days after pollination.

The ovules and ovaries of ‘Jingfurong1’ grew simultaneously, and the ovules filled the cavity in the seed coat 9 days after pollination. The seed’s coat changed from yellow to green 11 days after pollination, and the fruit expanded rapidly. After 14 days of pollination, the lotus seeds reached the maturity of fresh food. The size of the ovules of ‘Sijinggangshan’ did not change significantly, and the seed coat color changed to yellowish green seven days after pollination and did not change after that. After 9 days of pollination, the ovary also ceased to grow (Figure 1A). After artificial pollination, the embryo of ‘Sijinggangshan’ did not develop smoothly, and the ovule size did not change significantly. On the fourth day after pollination, the embryo sac began to atrophy, and the separation between the embryo sac and the inner integuments began. It completely atrophied 11 days after pollination (Figure 1B). The embryo of ‘Jinfurong 1’ developed smoothly.

### 2.2. Cytological Observation

**Figure 2:**
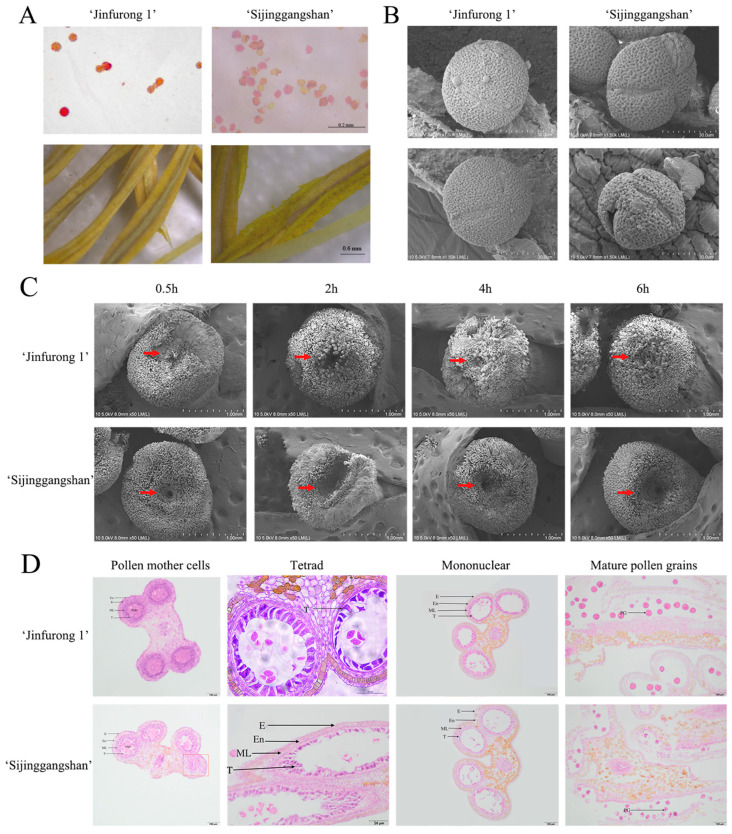
Comparison of pollen development of ‘Jinfurong 1’ and ‘Sijinggangshan’. (**A**) Pollen grain staining and powder dispersion of ‘Sijinggangshan’ and ‘Jinfurong 1’. (**B**) Pollen grain morphology of ‘Jinfurong 1’ and ‘Sijinggangshan’. (**C**) Pollen grain germination of ‘Sijinggangshan’ and ‘Jinfurong 1’ on stigma after artificial pollination. The stigma of the two cultivars was selected for observation at 0.5, 2, 4 and 6 h after artificial pollination. The red arrow points to the anther development of the stigma mouth. (**D**) Another development of ‘Jinfurong 1’ and ‘Sijinggangshan’. E, Epidermal layer; ML, middle layer; En, endothecium; T, tapetum; PMC, pollen mother cell.

TTC staining was an effective method to detect pollen grain viability. After TTC staining, the pollen grain staining rate of ‘Sijinggangshan’ was 11.2%, while that of ‘Jinfurong 1’ reached 52.1%. The pollen grain viability of ‘Jinfurong 1’ was significantly higher than that of ‘Sijinggangshan’. By observing the surface of mature anthers, we found that ‘Sijinggangshan’ had less pollen grain attached to the surface of the anthers, while ‘Jinfurong 1’ had a large amount of pollen grain stuck to the surface of the anthers (Figure 2A). The scanning electron microscopy (SEM) results showed that the pollen grain of ‘Jinfurong 1’ was full and close to the neat spherical shape rules, and the pollen wall structure was complete. Three long germination ridges showed uniform distribution on the surface of the pollen grain. Pollen grain morphological differences of ‘Sijinggangshan’ showed a high rate of deformity, with short germination pollen grain showing irregular distribution on the surface. Furthermore, some pollen grains appeared flat, and some pollen content overflowed from the germination ridge, with flocculent material existing outside the pollen wall (Figure 2B).

By detecting pollen grain germination of the two varieties after pollination, we found that a small amount of pollen grains had germinated on the stigma of ‘Jinfurong 1’ 0.5 h after pollination, and a large amount of pollen grain germinated at the stigma mouth 4 h after pollination (Figure 2C). On the other hand, until 6 h after pollination, only a minimal amount of pollen grains germinated on the stigma of ‘Sijinggangshan’, which could not meet plant fertilization needs (Figure 2C).

To determine the critical period of pollen grain sterilization, anther development of the two cultivars was observed. Compared with ‘Jinfurong 1’, the anther of ‘Sijinggangshan’ had the exact structure of the epidermis, endothecium, middle layer and tapetum. Still, the structure was relatively loose, and there were undifferentiated cell masses (Figure 2D) red box). At the tetrad stage, microspores of both species could be released normally, and tapetum began to degrade (Figure 2D). In the mononuclear stage, the tapetum cells of ‘Sijinggangshan’ showed lighter staining and severe degradation compared with ‘Jinfurong 1’ (Figure 2D). At the mature pollen grain stage, the two varieties’ tapetum, middle layer and inner were utterly degraded. The pollen grain of ‘Jinfurong 1’ had deep staining, and most were regular and round, while the pollen grain of ‘Sijinggangshan’ had light staining, and most were irregular in shape (Figure 2D), which was consistent with the morphology of the two varieties observed under the scanning electron microscope (Figure 2B), which proved that the pollen abortion of ‘Sijinggangshan’ may have occurred in the period of mononuclear pollen grains. It is speculated that it may be related to the abnormal tapetum.

### 2.3. Transcriptome Analysis

**Figure 3:**
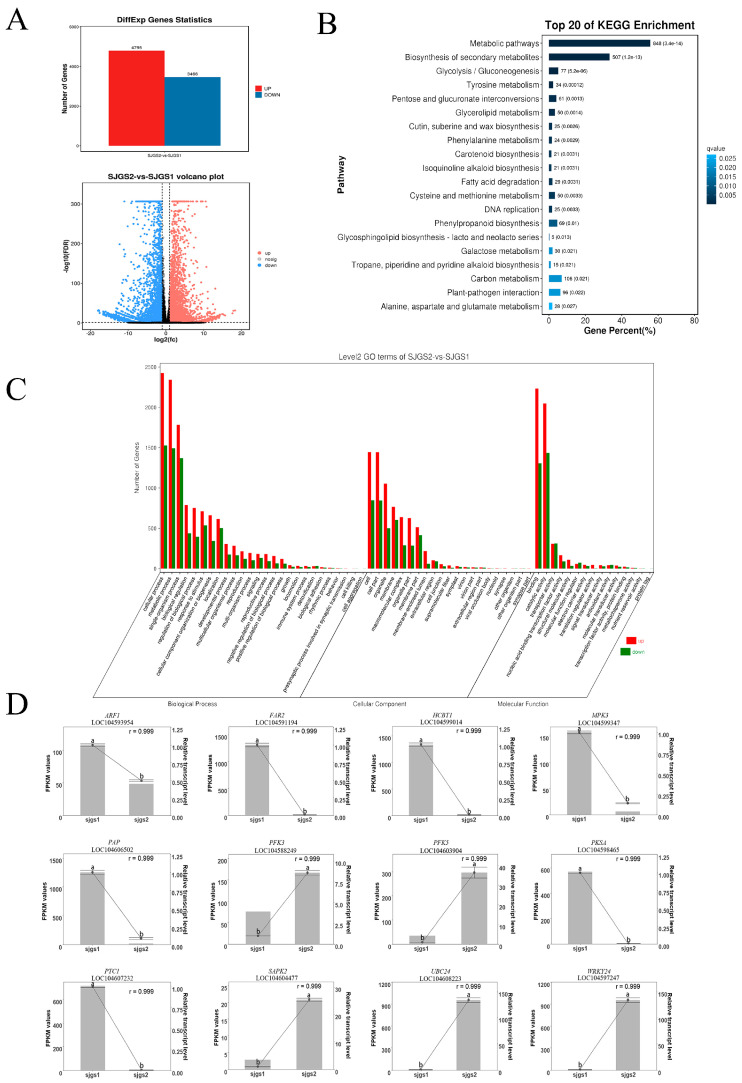
Transcriptome analysis of anthers during the critical period of abortion and after abortion in ‘Sijinggangshan’. (**A**) Histogram of differential genes and volcano diagram. (**B**) The top 20 KEGG pathways of differential genes. (**C**) Go classification map of differential genes. SJGS1 anthers during the critical period of abortion. SJGS2 anthers after completion of abortion. (**D**) qRT-PCR analysis of ARF1 auxin response factor 1 of 12 differential genes. FAR2 fatty acylCoA reductase 2, HCBT1 omega-hydroxypalmitate Oferuloyl transferase, MPK3 mitogen-activated protein kinase 3, PAP plastid-lipid-associated protein, PFK3 ATP-dependent 6-phosphofructokinase 3, PFK5 ATP-dependent 6-phosphofructokinase 3, PKSA type III polyketide synthase A, PTC1 PHD finger protein PERSISTENT TAPETAL CELL 1, SAPK 2 serine/threonine-protein kinase 2, UBC24 probable ubiquitin-conjugating enzyme E2 24, WRKY24 WRKY transcription factor 24. Black bars represent the mean FPKM values. Black dots represent the mean qRT–PCR values. Error bars indicate the SD for three biological replicates based on qRT–PCR, and shared letters indicate no statistically significant difference between the means (*p* > 0.05) as determined by ANOVA. The correlation coefficient was calculated by R with mean FPKM values and relative transcript levels generated by qRT–PCR.

To identify the essential genes affecting the abortion of lotus pollen grain, Illumina sequencing was performed before and after abortion of the ‘Sijinggangshan’ pollen grain. After analyzing the differentially expressed genes, 8261 significantly differentially expressed genes were obtained (Table S4). Among them, SJGS2 was upregulated with 4795 differential genes and downregulated with 3466 differential genes compared to SJGS1 (Figure 3A).

Differential genes were classified by GO annotation, and the classification results are shown in the following figure (Figure 3C). Among them are 26 groups in the biological process ontology and 21 in the cell component ontology. The process performs GO annotation classification on the differential genes, and the classification results are shown in the figure above. Among them are 26 groups in biological process ontology, 21 in cell component ontology, and 15 in molecular function ontology. The most differentially distributed genes in biological processes were cellular processes (3952), metabolic processes (3834) and single tissues (3152). Metabolic processes include glycolysis, the gluconeogenesis pathway, secondary metabolite synthesis pathway, and phenylpropanoid metabolism pathway. The most extensive distribution of differential genes in cellular fractions was in cells (2292) and organelles (2289). The large proportion of genes involved in cells and organelles indicates that there were more genes involved in ribosomes, mitochondria, chloroplasts, Golgi and endoplasmic reticulum, further indicating that ‘Sijinggangshan’ pollen grain infertility was related to the development of organelles. In terms of molecular function, the differentially expressed genes were mainly distributed in binding (3539) and catalytic activities (3483). That is, various enzymes accounted for a large proportion, indicating that multiple enzymes were involved in the abortion of the ‘Sijinggangshan’ pollen grain.

Significant KEGG enrichment can show the most important pathways involved in the screened genes. We conducted KEGG enrichment analysis for the differential genes (Figure 3B), and 135 pathways were enriched (Table S6). More differential genes were enriched in metabolic pathways and secondary metabolite synthesis, which were 808 and 507, accounting for 55.68% and 33.29%, respectively. Some other differential genes were enriched to courses related to cellular PCD, such as RNA degradation, ubiquitin-mediated protein hydrolysis, and phagosome, with 30, 34, and 24, respectively, accounting for 1.97%, 2.23%, and 1.58%. Therefore, metabolic disorders, abnormal secondary metabolite synthesis, programmed cell death and so on may cause the fertility of ‘Sijinggangshan’. Plant hormones also have a significant impact on plant growth and development. One hundred differential genes were enriched in plant signal transduction pathways, accounting for 6.57%, indicating that abnormal signal transduction of plant hormones may lead to decreased pollen grain fertility.

We first screened 115 differentially expressed genes that may be related to pollen grain abortion (Figure S1) and then randomly selected 12 genes for qRT-PCR validation. These genes are mainly involved in plant signal transduction, energy supply and programmed cell death. The results showed that the expression trends of almost all genes were consistent with the sequencing data (Figure 3D).

### 2.4. Expression Pattern of NnPTC1

**Figure 4:**
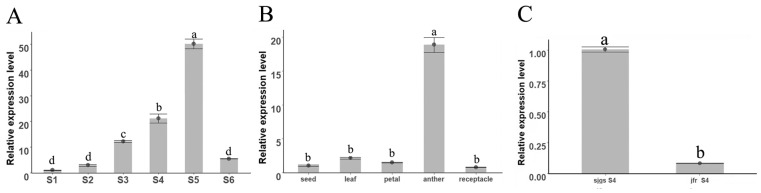
Expression analysis of *NnPTC1* in ‘Jinfurong 1’. (**A**) Expression of *NnPTC1* in lotus anthers at different pollen development stages. (**B**) Expression of *NnPTC1* in different parts of lotus. (**C**) Expression of *NnPTC1* in ‘Sijinggangshan’ and ‘Jinfurong 1’ during pollen development at the mononuclear stage. S1, Pollen mother cell stage; S2, meiotic phase; S3, tetrad period; S4, Mononuclear period, S5, Binucleate period; S6, pollen grain maturity; sjgs, ‘Sijinggangshan’; jfr, ‘Jinfurong 1’. Values are the means ± SE of three independent experiments with at least three replicates for each. According to Tukey’s multiple test, bars marked with different letters are significantly different at *p* < 0.05.

Since the function and transcriptional regulation of *NnPTC1* during another development in lotus remains unclear, we first investigated the expression pattern of *NnPTC1*. In the anthers of ‘Jinfurong1’, the expression of *NnPTC1* was significantly higher during pollen binucleation than at other stages of pollen development (Figure 4A). In lotus, *NnPTC1* was expressed considerably in anthers, while there was no significant difference in the expression of other parts (Figure 4B). In anthers at the mononuclear stage of pollen development (critical stage of abortion), the expression of *NnPTC1* in ‘Sijinggangshan’ was significantly higher than in ‘Jinfurong 1’ (Figure 4C).

### 2.5. Phylogenetic Tree and Sequence Analysis

**Figure 5:**
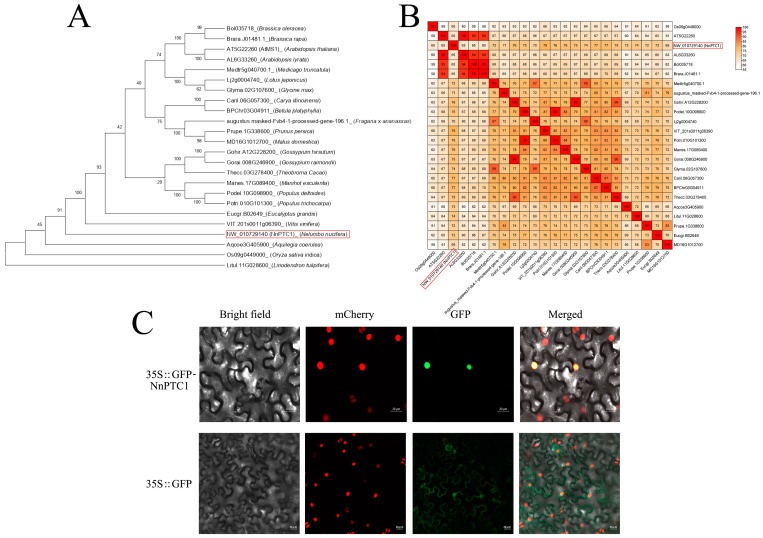
Basic information of *NnPTC1*. (**A**) Evolution tree of PTC1 homolog genes. *NnPTC1* in red box. (**B**) Comparison of *NnPTC1* and homolog proteins in the red box. For details of *NnPTC1*, please see Supplementary File S1. (**C**) *NnPTC1* subcellular localization of GFP, green fluorescence protein; mCherry, red fluorescent dye; Merged, overlay plots.

The protein sequences of Arabidopsis AtPTC1/AtMS1 (AT5G22260), indica OsPTC1 (Os09g0449000) and lotus *NnPTC1* (NW_010729140) were blasted in the phytozomeprogram, and the PTC1 proteins of 24 species were identified to construct an evolutionary tree. Phylogenetic tree analysis showed that cruciferous vegetables such as cabbage, Chinese cabbage, *Arabidopsis thaliana* and lute leaf shared a class with the PTC1 protein; leguminous thistles of alfalfa, lotaustralin root, soybean shared class with the PTC1 protein; Rosaceae strawberry, peach, and apple shared a class with the PTC1 protein; and mallows such as upland cotton, Raymond’s cotton and cocoa tree shared a class with the PTC protein. The *NnPTC1* gene of lotus belongs to a separate class (Figure 5A). The average similarity of the *NnPTC1* gene protein sequence in different species was 72.75%, the average similarity of the *NnPTC1* protein sequence with the other 23 members was 72.16%, and the highest similarity with grape (VIT_201s0011g06390) was 78.93%. Conversely, the lowest similarity with Arabidopsis AtPTC1 was 65.07% (Figure 5B).

### 2.6. Subcellular Localization of NnPTC1

To explore the intracellular localization of the *NnPTC1* protein, the vector pFast-R05-NnPTC1 fused with GFP tag was constructed, transferred into Agrobacterium GV3101 and injected into the leaves of transgenic tobacco with nuclear localization. The subcellular localization of green fluorescence fusion protein was detected by confocal microscopy. It was observed that 35S:: *NnPTC1*-GFP was localized in the nucleus, while 35S:: GFP was distributed in the cell membrane and nucleus. (Figure 5C).

### 2.7. The Seed Setting Rate of Overexpressing NnPTC1 Was Down-Regulated

**Figure 6:**
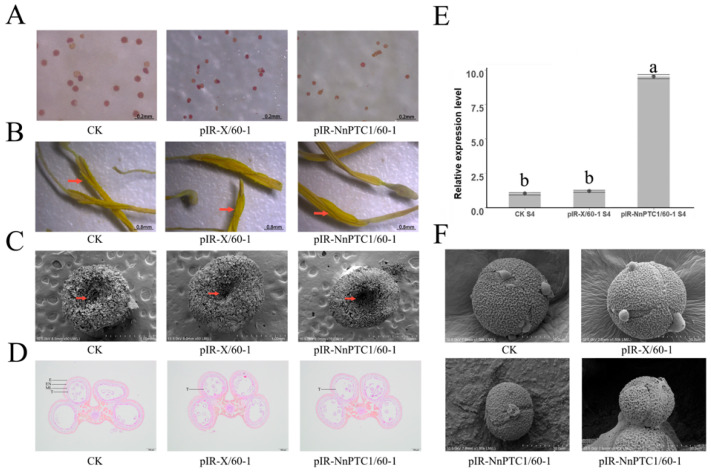
Pollen development of ‘Jinfurong 1’ after transient transformation of *NnPTC1* overexpression. (**A**) Pollen grain staining of ‘Jinfurong1’ after transient transformation. (**B**) Pollen grain of ‘Jinfurong 1’ spread on the anther surface after transient transformation. Red arrows point to the anther surface. (**C**) Pollen grain germination on stigma of ‘Jinfurong 1’ after artificial self-pollination after transient transformation, red arrow points to stigma mouth, and all samples were taken 6 h after artificial pollination. (**D**) Structure of anther of ‘Jinfurong1’ at microspore mononuclear stage after transient transformation. E, epidermal layer; ML, middle layer; En, endothecium; T, tapetum. (**E**) Expression of *NnPTC1* after transient transformation. (**F**) Pollen grain morphology of ‘Jinfurong 1’ after transient transformation.

To further the role of *NnPTC1* in pollen grain fertility, we selected ‘Jinfurong 1’ lotus flowers with basically the same growth and carried out the infection experiment of the twin vector of overexpression virus (pIR-NnPTC1) with a transient infestation at 7-day intervals. After the overexpression treatment of ‘Jinfurong 1’, anthers at the mononuclear stage were taken for qRT-PCR detection. The expression of *NnPTC1* in overexpression-treated plants was found to increase significantly. In contrast, the expression of empty vector-treated plants had no significant difference compared with CK (Figure 6E). After the transient transformation of *NnPTC1*, ‘Jinfurong 1’ was strictly self-crossed, and the fruitbearing was counted. Compared with the blank control, the seed setting rate of ‘Jinfurong 1’ decreased significantly after overexpression of *NnPTC1*, and the decrease rate reached 40%. However, there was no significant change after overexpressing the empty vector. In the case of open pollination, there was no significant difference in seed setting rate between the transiently transformed lotus and the blank control.

After overexpression of *NnPTC1*, the pollen grain viability and powder distribution of ‘Jinfurong 1’ were statistically observed. The pollen grain viability of the blank control reached 51%, while the pollen grain viability of the overexpression treatment was only 31%, significantly different from the blank control. Stereomicroscopical observation of the anther surface showed that compared with the blank control, the pollen grain spread on the anther surface was significantly reduced after *NnPTC1* overexpression (Figure 6B). After *NnPTC1* overexpression, the pollen grain morphology was observed by scanning electron microscopy. The pollen grain of blank control was full and nearly regular spherical in shape. There were three sprouting ridges, which were long and linear, and the pollen grain wall structure was complete. After overexpression of *NnPTC1*, the pollen grain morphology of lotus changed significantly compared with the blank control. The pollen grain is irregular in shape, and the sprouting ridge appears curved in ratio A. There were flocculent materials on the surface of the pollen grain, and the phenomenon of adhesion between the pollen grains appeared (Figure 6F). Scanning electron microscopy was used to observe the germination of lotus pollen grain on the stigma after transient transformation of *NnPTC1*. The pollen grain of blank control germinated in large quantities near the stigma mouth. Compared with the blank control, the amount of pollen grain germinating on the stigma was significantly reduced after *NnPTC1* overexpression (Figure 6C). Paraffin sections were used to observe the structure of each part of the transiently transformed anther from microspore to the mononuclear stage. The tapetum of the blank control remained intact. Compared with the blank control, the tapetum of the blank control after *NnPTC1* overexpression showed severe degradation in the mononuclear stage, the tapetum was separated from the drug chamber wall, and the tapetum structure was incomplete and interrupted. (Figure 6F). These results proved that *NnPTC1* caused excessive degradation of the tapetum of lotus, resulting in decreased pollen grain fertility.

## 3. Discussion

### 3.1. Cytological Mechanism of Low Seed Setting Rate in the Double Lotus

The sexual reproduction process of plants is complex and delicate and is subject to various internal and external environmental influences. Therefore, the failure of plants to develop into mature and full seeds in the process of sexual reproduction is called seed abortion [25]. Embryo abortion is a typical phenomenon of seed embryo abortion. Embryo abortion is a process that occurs after pollination and fertilization of plants. As the embryo grows from zygote development to the mature embryo formation stage, its development stops at a particular stage due to various reasons. In horticultural plants, there have been a large number of relevant studies on embryo abortion, which declare that hormone content disorder [26] and endosperm development [27] are related to the secretion of quinones [28]. Our study found that the ovule of ‘Sijinggangshan’ had no apparent signs of development. Through section observation, it was found that the embryo sac had begun to atrophy on the fourth day after fertilization, indicating that fertilization may not have been completed to form zygotes rather than embryo abortion.

A smooth fertilization process of plants is the key factor affecting the seed-setting rate. In previous studies of *Arabidopsis thaliana*, FERONIA receptor kinases were found to have dual roles in ensuring sperm delivery and preventing polyspermatozoa. *FERONIA* can alter the ovate condition at the arrival of the first pollen tube to remove the pollen tube and prevent the late pollen tube from entering and penetrating the female gametophyte, thus avoiding multiple fertilization [29,30]. By scanning electron microscopy, we observed that a large amount of pollen grain germinated smoothly on the stigma surface of ‘Jinghurong 1’ after artificial fertilization. However, the pollen tube reached the stigma, and ‘Sijinggangshan’ stigma surface germination of the pollen grain quantity was small and could not meet the requirements of fertilization, which supported our conclusion that the low fruiting rate of Sijinggangshan’ was not caused by embryo abortion midway. According to previous studies, abnormal pollen grain morphology [31,32] will affect pollen grain vitality, thus reducing plant seed setting rate. Japanese scholars obtained two CMS types of sterile lines after continuous backcrossing by hybridization of the mutant Mannera Chinensis. They cultivated mannera solanum, one of which was anther indehiscent, and the other was low pollen grain type [33]. The results indicated that the low amount of anther powder was also a critical factor causing male sterility. In our study, we found that the pollen grain viability of ‘Sijinggangshan’ was significantly lower than that of ‘Jinfurong 1’. The anther surface of ‘Jinfurong 1’ was scattered with a large amount of pollen grain, while the anther surface of ‘Sijinggangshan’ was rarely distributed with pollen grain. Therefore, it was speculated that the anther surface of ‘Sijinggangshan’ had less pollen grain and low pollen grain vigor, resulting in its low seed-setting rate.

Pollen development is a highly complex process involving various cellular changes and physical and chemical reactions. The anther wall of plants is divided into four layers: epidermis, inner wall, middle layer and tapetum, among which the tapetum plays a crucial role in pollen grain development. Tapetum cells duly enter the PCD process and release many nutrients and structural substances such as sugars, lipids and proteins into the anthers to ensure the normal development of microspores and successful pollination [34]. Tapetum starts the PCD process in advance, and this abnormal tapetum degradation that is out of sync with microspore development may be one of the main reasons for pollen grain abortion [35]. Much sporopollenin released by tapetum degradation is transported to the outer walls of pollen mother cells, forming a spherical grain wall with regular morphology and complete structure [36]. The early degradation of tapetum may result in an irregular pollen outer wall due to the inability to release lipid substances needed to form sporophytes, ultimately leading to pollen abortion [37]. In our study, we found that the pollen outer wall of ‘Sijinggangshan’ is irregular, and the pollen malformation rate is high, which may be the main reason for its poor fertility. Previous studies on male sterility in grape cultivars ‘Wink seeding-12’and ‘Zhongshanhongearly’ tapetum suggest degradation at a high seeding rate that leads to low nutrient level to be the cause of pollen sterility [38,39]. Our study also found that the tapetum of ‘Sijinggangshan’ had been significantly degraded during the mononuclear stage, which may be the critical factor leading to pollen sterility.

### 3.2. Gene Mining of Low Seed Setting Rate in the Double Lotus

Studies on the molecular mechanisms of pollen development have focused on model plants such as rice and Arabidopsis. Genes regulating the development characteristics of tapetum have been found in *Arabidopsis thaliana*. *SPOROCYTELESS*(*SPL*)/*NZZ* (*NOZZLE*). Mutations in *SPL/NZZ* genes arrest the development of the tapetum, anther and spore mother cell primordia and fail to form anther [40]. After the formation of tapetum, *DYT1-TDF1-AMS-MYB80-MS1*, a genetic regulatory pathway, may play an important role in the development of the tapetum and have an important impact on pollen development [41]. SlHB8 in the HD-Zip III transcription factor family can negatively regulate the *DYT1-TDF1-AMS-MYB80* genetic pathway and participate in the development and degradation of tapetum [42]. *PERSISTENT TAPETAL CELL 1* (*PTC1*) is a homologous gene of *MS1* in rice, and its function is similar to *MS1*. ptc1 mutants show male sterility, and the membrane and organelles of the tapetum remain intact without signs of rupture and apoptosis. The tapetum is extensively proliferated and enters into the anther compartment [22]. We found the expression of many genes related to the programmed death of tapetal cells in transcriptome files, such as MSTRG.12778 (*AMS*) ncbi_104588551 (*MYB80*) and ncbi_104607232 (*PTC1*). The expression of a large number of genes related to programmed cell death increased, such as ncbi_104610637, ncbi_104601719, ncbi_104589106, ncbi_104611838, etc. The gene ncbi_104607232 is predicted to be the gene encoding the PTC1 protein in lotus, and its expression level is significantly increased compared with that in the late stage of abortion. We speculate that the overexpression of ncbi_104607232 may lead to the excessive degradation of tapetum cells, thus decreasing pollen fertility.

In addition to the above key genes, some genes related to energy supply and material metabolism were also differentially expressed during the critical period of abortion, such as ncbi_104613189, ncbi_104603904, ncbi_104588249, etc. In pollen development, the disorder of energy supply and material metabolism will also cause pollen abortion [43,44]. Furthermore, many genes related to hormone signal transduction were also differentially expressed, such as ncbi_104610541, ncbi_104599112 and ncbi_104592780. Plant hormones play a crucial role in plants’ reproductive growth. In previous studies, mutants of genes involved in plant signal transduction produced significantly less regular pollen grains [45,46].

### 3.3. NnPTC1 Promotes Pollen Abortion of Double Lotus

Proteins containing the PHD domain can be widely involved in plant life activities as transcription factors. For example, the PHD family gene PTC1 is an essential gene for determining the fate of tapetum cells and pollen grain formation in monocot rice and for regulating the programmed development and degradation of the villus layer during anther development in rice. Loss of PTC1 function leads to uncontrolled proliferation and swelling of tapetum cells, delayed DNA breakage, abnormal pollen wall development, and complete male sterility [22].

In this study, we used qRT-PCR to analyze the expression of *NnPTC1* in lotus. Moreover, we found that the expression of *NnPTC1* in ‘Sijinggangshan’ was significantly higher than that in ‘Jinfurong 1’ during the mononuclear stage of microspore development, indicating that *NnPTC1* may be an essential gene affecting the vast difference in tapetum structure between the two varieties. Further study on the expression characteristics of *NnPTC1* showed that the expression of *NnPTC1* in the binucleate phase was significantly higher than that in the mononuclear phase. The lower expression of *NnPTC1* in the mononuclear phase may be the reason for the tapetum cells in the mononuclear phase of ‘Jinfurong 1’ remaining intact. Analysis of the expression level of *NnPTC1* in different parts of the lotus showed that the gene was strongly expressed in anthers. In contrast, the expression level was low in other parts, indicating that *NnPTC1* was mainly involved in life activities related to pollen development.

PTC1 shares homology with many proteins that contain PhD-finger motifs in animals, yeast, and higher plants [47]. Phylogenetic analysis showed that Arabidopsis MS1, poplar PtMS1, and rice PTC1 form an independent group within the whole family [48], suggesting that PTC1 and its homologs may have conserved roles in plant reproductive development. The sequences of *NnPTC1* and its homologous proteins were compared, and the results showed that the evolutionary clustering of the PTC1 gene was consistent with the phylotaxonomic relationship and evolutionary process of plants, indicating that the protein was relatively conserved in the evolutionary process. In the process of comparison, it was found that the protein similarity with grape (VIT_201s0011g06390) reached 78.93%, which was mutually verified with the previous finding that the excessive degradation of tapetum in the mononucleic stage resulted in grape abortion [38].

In rice, PTC1 driven by the heterologous transformation of MS1 promoter can rescue pollen wall development and pollen grain fertility of homozygous MS1 mutants in Arabidopsis, indicating a conserved role in regulating programmed underdevelopment in monocot and dicot plants [22]. In this study, we isolated *NnPTC1* from lotus and treated ‘Jinfurong1’ with a transient transformation system. After overexpression of *NnPTC1*, the expression of *NnPTC1* in anthers at the mononuclear stage was significantly increased compared with the blank control. After overexpression of *NnPTC1*, the seed-setting rate of ‘Jinfurong 1’ decreased significantly. In contrast, the seed-setting rate did not change significantly under the condition of open pollination, which indicated that overexpression of NnPTC1 significantly affected the seed-setting rate of lotus flowers. The effect of pistile development and embryo abortion on the seed-setting rate of lotus flowers could be excluded. Subsequently, the properties of pollen grain after transient transformation were studied, and it was found that after overexpression of *NnPTC1*, the pollen grain viability of ‘Jinfurong 1’ was significantly reduced. The amount of scattered powder on the anther surface was also greatly reduced. The following scanning electron microscope observation confirmed the hypothesis. The observation results showed that after the overexpression of *NnPTC1*, the amount of pollen grain germinating on the stigma of hand-pollinated lotus flowers was less, which was similar to the observation of ‘Sijinggangshan’ in the early stage. Scanning electron microscopy was used to observe pollen grain morphology. It was found that after *NnPTC1* overexpression, the pollen grain malformation rate of ‘Jinfurong 1’ increased, and there was flocculent material on the surface, which made the pollen grain adhere to each other. The anthers tapetum can secrete callose enzyme and dissipate the callose wall between the microspores [49], which may be due to the excessive degradation of the tapetum, resulting in the insufficient release of callose enzyme and incomplete degradation of callose. After overexpression of *NnPTC1*, the tapetum of ‘Jinfurong 1’ was degraded more severely than that of the blank control. The early programmed death of tapetum cells leads to the early degradation of the tapetum, which can affect pollen development, activity and powder dispersion [50,51,52]. In fertile plants, the programmed death of tapetal cells occurs gradually, and the unnatural and explosive programmed death of tapetal cells may be the direct cause affecting plant fertility. Overexpression of *NnPTC1* leads to excessive degradation of the tapetum of lotus, which is the direct reason for the decrease in pollen grain fertility. The PTC1 gene encodes a zinc-finger transcription factor specifically expressed in tapetum cells and microspores. This transcription factor interacts with OsMADS15 and TIP2 protein and regulates EAT1 gene expression by coordinating TDR protein through the TIP2 gene. Furthermore, it regulates the degradation process of tapetum and the formation of the pollenouter wall [22,53]. Subcellular localization of *NnPTC1* was performed and found localized in the nucleus, laying a foundation for screening interacting proteins in the next step.

## 4. Materials and Methods

### 4.1. Experimental Materials

The lotus varieties used in this experiment were planted at Baima Teaching and Scientific Research Base of Nanjing Agricultural University (31° 95′ N, 118° 85′ E), which had a low double self-pollination and seed-setting rate (Sijinggangshan) and high double self-pollination and seed-setting rate (Jinfufang 1).

### 4.2. Artificial Pollination

From June to August 2021, well-developed buds were selected from robust plants and isolated by manual emasculation and bagging. Hand pollination was carried out on a sunny day, and pollination was carried out when flowers opened. A pollen grain of the two varieties was taken from 5:00 to 6:00 in the morning, and the anthers were placed on the stigma to be pollinated, bagged and isolated, strictly self-pollinated, and labeled. Thirty flowers were pollinated for each cultivar, of which 10 were used to count the self-set rate after 15 days, and 20 were used to study pollen grain germination on the stigma and the development of embryos.

### 4.3. Pollen Grain Viability and Quantity of Loose Powder

First, 0.5% TTC solution (weight 0.5 gTTC (Solarbio, Beijing, China) was prepared and dissolved in 100 mL distilled water) in a brown drop bottle, and kept out of light for later use. The pollen grains of the two varieties were collected in PCR tubes at 6:00 a.m. on a sunny day, and 200 μL TTC solution was added to the PCR tubes, mixed by shaking, and then placed in an incubator at 37 °C to avoid light for 15 min. Microscopic observation on the slides with 2–3 drops of staining solution showed that the pollen grain dyed red was the most vigorous, followed by the light red, and the unstained pollen grain was the worst. Three replicates were set up for each treatment, and three fields of view were observed for each replicate.

On sunny days, anthers with mature pollen grains were collected at 6 a.m., placed on slides, observed and photographed with a stereoptometer (M165FC, Leica, Wetzlar, Hesse-Darmstadt, Germany).

### 4.4. Pollen Grain Morphology Observation

The mature pollen grain of the bagged lotus flowers was collected and fixed with 2.5% glutaraldehyde (0.1 mol/L phosphate buffer, pH = 7.2) (Macklin, Shanghai, China) for more than 48 h, then dehydrated, dried and sprayed with gold. Then, the fixed materials were observed and photographed under a scanning electron microscope (SU8010, HITACHI, Tokyo, Japan).

### 4.5. Observation of Pollen Grain Germination on Stigma

Three pollinated lotus flowers were taken at 0.5, 2, 4 and 6 h of pollination, and the stigma was quickly removed with tweezers and fixed in 2.5% glutaraldehyde (0.1 mol/L phosphate buffer, pH = 7.2) glutaraldehyde for more than 48 h. The materials were observed and photographed under a SU8010 scanning electron microscope (SU8010, HITACHI, Tokyo, Japan).

### 4.6. Embryonic Development

At 2, 4, 8, 11 and 15 d after manual pollination, three pollinated carpels were collected and placed in 70%FAA (70% alcohol:aceticacid:formaldehyde solution = 90∶5∶5) fixation solution (Phygene, Fuzhou, Fujian, China) and stored at 4 °C. Sections with a thickness of 8 10 μm were stained with eosin. The sections were observed and photographed under a stereomicroscope in Germany (M165FC, Leica, Wetzlar, Hesse-Darmstadt, Germany).

One fruit was taken, the outer skin was removed, and the embryo development was observed and photographed using Canon 600D (Canon, Tokyo, Japan).

### 4.7. Pollen Development

At 0, 2, 4, 6, 8, 10 and 12 d after bud appearance, flower buds of three different plants were selected and fixed with 70% FAA (70% alcohol∶aceticacid:formaldehyde solution = 90:5:5) fixation solution (Phygene, Fuzhou, Fujian, China) and stored at 4 °C. Anthers were dissected from our fixed bud species, sectioned using conventional paraffin sectioning techniques, 8–10μm thick, stained with eosin and hematoxylin, and observed and photographed under a Zeiss microscope.

### 4.8. Transcriptome Sequencing

According to the results of previous cytological studies, the lotus variety ‘Sijinggangshan’ was identified as the transcriptome sequencing cultivar. Anthers before abortion were selected and named SJGS1, and anthers after abortion were named SJGS2. The two materials were used for transcriptome sequencing

Total RNA was extracted using a Trizol reagent kit (Invitrogen, Carlsbad, CA, USA) according to the manufacturer’s protocol. The ligation products were sequenced using Illumina HiSeq2500 by Gene Denovo Biotechnology Co. (Guangzhou, China). The lotus genome (http://www.ncbi.nlm.nih.gov/genome/?term=nelmbo+nucifera, accessed on 15 April 2020) was used for reading mapping. Expression values were normalized in FPKM (fragments per kilobase of exon per million fragments mapped). RNA differential expression analysis was performed by DESeq2 [54] software between two different groups (and by edgeR [55] between two samples). The genes/transcripts with false discovery rate (FDR) parameters below 0.05 and absolute fold change ≤ 2 were considered differentially expressed genes/transcripts. Transcriptome data were deposited to the NCBI with accession number PRJNA868584. Cluster Profiler was used to perform GO functional enrichment analysis and KEGG pathway enrichment analysis on the differential gene sets.

### 4.9. qRT-PCR Analysis of DEGS and NnPTC1

RNA-Seq data were verified by qRT-PCR analysis. Primer pairs for each gene were designed using Primer Premier 5.0 software (Supplementary Table S1). qRT-PCR was performed on a LightCycler480 II (Roche, Rotkreuz, Switzerland) instrument using Fast SYBR Green Master Mix (B639271, BBI). The reaction protocol was as follows: 95 °C for 3 min, followed by 45 cycles of 95 °C for 5 SEC and 60 °C for 30 SEC. The relative expression levels of each target gene were calculated by the 2^−ΔΔCt^ method. To ensure the libraries’ reliability, we randomly selected 12 DEGs from each library and validated the data by qRT-PCR. To research the expression of *NnPTC1* in lotus after injection, we performed qRT-PCR analysis. All gene primers were designed using Primer Premier 5.0 (Premier Biosoft International, Palo Alto, CA, USA);the reference gene *Nnactin* [56] was used to normalize the expression levels using the 2^−ΔΔCT^ method56]. The sequences of all primers, including *Nnactin*, are listed in Table S2. The total RNA from a lotus was prepared using a Vazyme reagent kit (VAZYME, Nanjing, Nanjing, Nanjing) China) according to the manufacturer’s procedure. Reverse transcription was carried out with the Vazyme (VAZYME, Nanjing, China) reagent kit, and qRT-PCR analysis was performed using ChamQ SYBR qPCR Master MIX (VAZYME, Nanjing, Nanjing, Nanjing) China) with the Quant StudioTM Real-Time PCR system (Bio-Rad, Hercules, CA, USA). Relative expression levels of the DEGs were calculated by the 2 ^∆∆Ct^ method [57]. Three biological replicates were used for all qRT-PCR experiments.

### 4.10. NnPTC1-Based Transformation

‘Jinfurong1’ was used as the lotus cultivar for *NnPTC1* cultivar to perform gene expression or silencing of the lotus via modified tomato yellow leaf curl virus (TYLCV)-based geminivirus vector system (IL-60-BS/IR), which is a nontransgenic universal vector system used for gene expression and silencing [58,59]. *NnPTC1* overexpression vector (pIR-NnPTC1) was injected into the two-month-old lotus leaves with IL-60-1 plasmid at a ratio of 1:1 (800 ng/100 uL). The phenotypes, gene expression level and *NnPTC1* were analyzed 10 days after infection. Three biological replicates were used in all experiments.

### 4.11. Gene Sequence Analysis

The total RNA from lotus was prepared using a Vazyme reagent kit (VAZYME, Nanjing, China)according to the manufacturer’s procedure. The target gene’s coding sequence (CDS)was amplified from the cDNA template using primers designed according to the transcriptome sequence (ncbi_104607232). *NnPTC1* protein sequences were downloaded from phytozome (https://phytozome-next.jgi.doe.gov accessed on 1 October 2022)and subjected to phylogenetic analysis using MEGA X. Sequence alignment was conducted using DNAMAN 5.2.2 software for visualization.

### 4.12. Subcellular Localization of NnPTC1

The LR reaction was used to recombine the pENTR 1A-NnPTC1 plasmid with the binary vector pFAST-R05 (p35S::GFP) to generate a fusion plasmid (pFAST-R05-NnPTC1) harboring the p35S::GFP-NnPTC1 construct. As previously described, the pFAST-R05-NnPTC1vector was introduced into the nuclear-marked (marked by mCHERRY red) tobacco using Agrobacterium-mediated genetic transformation. After injection, tobacco was cultured for 24 h in the dark and then transferred to the culture chamber under extended daylight (light/dark =16/8 h) for 2 days. GFP activity was determined using confocal microscopy (Carl Zeiss AG, Oberkochen, Germany).

## 5. Conclusions

A low self-fertilization rate significantly affects cross-breeding and the promotion of double lotus. Therefore, it is necessary to fully understand the mechanism involving the self-fertilization rate and pollen grain activity of double lotus to develop a method to improve the self-fertilization rate of double lotus. In this study, we found that low pollen grain vitality was the main reason for the low self-fertilizing rate of double lotus, and scanning electron microscopy (SEM) confirmed that early tapetum degradation in the mononucleosis stage directly led to a decrease in pollen grain vitality. In addition, transcriptome sequencing screened the transcription factor NnPTC1, and transient transformation technology identified the function of the critical gene *NnPTC1*. Therefore, we established a molecular model with *NnPTC1* as the core component to regulate the pollen grain viability and self-fertilizing rate of double lotus. This study can improve our understanding of the cytological and molecular regulatory mechanisms of the low pollen grain viability of double lotus resulting in a decrease in the self-fertilizing rate, and it lays an excellent theoretical foundation for the next step of cultivating lotus varieties that use both food and appreciation.

## Figures and Tables

**Table 1:** Self-breeding of ‘Jinfurong 1’ after transient transformation.

	Number of Stigmas in Open Pollination	Seed Number in Open Pollination	Seed Setting Rate by Open Pollination (%)	Stigma Number of Artificial Pollination	Seed Number in Artificial Pollination	Seed Setting Rate by Artificial Pollination (%)
CK	124.3 ± 5.6 a	100.4 ± 3.3 a	80.77 ± 1.12 a	133.1 ± 5.1 a	81.2 ± 3.12 a	61.00 ± 2.14 a
pIR-X	128.2 ± 4.3 a	104.3 ± 2.8 a	81.48 ± 2.12 a	145.1 ± 6.2 a	92.3 ± 4.3 a	63.61 ± 2.12 a
pIR-NnPTC1	121.3 ± 5.7 a	99.27 ± 4.2 a	81.84 ± 3.12 a	137.1 ± 3.5 b	48.2 ± 6.2 b	35.15 ± 3.12 b

CK, blank control; pIR-x, overexpression of empty vector treatment; pIR-*NnPTC1*, NnPTC1 overexpression treatment; Values (a,b) represent mean ± SE (n = 3). Significant differences were analyzed using Tukey’s multiple range test.

## Data Availability

The data that support the findings of this study are available from the corresponding author upon reasonable request.

## Supplementary Materials

The following supporting information can be downloaded at: https://www.mdpi.com/article/10.3390/plants12020387/s1, Figure S1: Heatmap of 135 differentially expressed genes; File S1: PTC1 homologous protein sequence; Table S1: Self-Pollinated of ‘Jinfurong 1’ and ‘Sijinggangshan’; Table S2: Primer sequences used in the study; Table S3: all.genes.expression.annot; Table S4: DEGs in SJGS1-VS-SJGS2; Table S5: SJGS2-vs-SJGS1.Go analysis of differentially expressed genes; Table S6: Information on 135 differentially expressed genes; Table S7: SJGS1-vs-SJGS2.KEGG path.
