# Supplementary material for: Cytological and Molecular Mechanism of Low Pollen Grain Viability in a Germplasm Line of Double Lotus
Source: Plants (Basel). 2023 Jan 13;12(2):387. doi: 10.3390/plants12020387 (PMC9867118; doi:10.3390/plants12020387)
Supplement: Supplementary file 1 [file plants-12-00387-s001.zip › ╕╜╝╙╬─╝■/Table S1 Self-Pollinated of 'Jinfurong 1' and 'Sijinggangshan'.pdf]

Table S1 Self-Pollinated of 'Jinfurong 1' and 'Sijinggangshan'

	Number of stigmas in open pollination	Seed number in open pollination	Seed setting rate by open pollination	Stigma number of artificial pollination	Seed number in artificial pollination	Seed setting rate by artificial pollination
'Jinfurong 1'	135.3±5.6a	105.4±4.3a	80.15%±3.62a	142.5±5.9a	90.4±3.9a	63.33%±3.14a
'Sijinggangshan'	127.2±4.3a	19.3±2.3b	15.17%±1.07b	132.3±7.3a	9.3±1.2b	7.09%±0.92b
