# Supplementary material for: Cytological and Molecular Mechanism of Low Pollen Grain Viability in a Germplasm Line of Double Lotus
Source: Plants (Basel). 2023 Jan 13;12(2):387. doi: 10.3390/plants12020387 (PMC9867118; doi:10.3390/plants12020387)
Supplement: Supplementary file 1 [file plants-12-00387-s001.zip › ╕╜╝╙╬─╝■/Table S2 Primer sequences used in the study.pdf]

TABLE S2. Primer sequences used in the study.

Unigene ID	Symbol	Forward primer	Reverse primer
ncbi_104599347	MPK3	ACAACTTCCTCCACATCCTCG	CTCCTTCATTTGCTCTTCCACT
ncbi_104607232	PTC1	GAAGGATGGTAGAGGAGGGGAT	ATGGTAGGGAAGGAAGGAGGAC
ncbi_104608223	UBC24	TACGTTCTACGAAACCCACCC	TTCTTGCTCAGCCACTCCAC
ncbi_104597247	WRKY24	GCGTTAGACCACCAATAACAGC	TCAAGAAACAAGTCATCCCCG
ncbi_104603904	PFK5	CGAGGAGTTTGCAGATGGTTAT	CCAGCAGATGTAGGTGAGGAGTA
ncbi_104588249	PFK3	CATTCAGGACCGGGGTATTAA	GGATTCCAGCAACTGCAACTT
ncbi_104598465	PKSA	CAAGGACATAGCCGAGAACAAC	ACAGAGCAGCACCAACGAGA
ncbi_104606502	PAP	CGTGGGCTTATTCCTGCTGT	TGGTGCTCAAGGTTGTGGTG
ncbi_104604477	SAPK2	TCATTTTCTCGGTGGACGG	TTGGCATTGAAGTTGGCG
ncbi_104593954	ARF1	CCAGTTTTACCTCCTCCGACA	GTGACTGAGGAGAAGAGGGATGT
ncbi_104599014	HCBT1	TACCCAAGCCATCTACAACGA	AGAGGAGAATGAGGAAGTTACTGA<br>A
ncbi_104591194	FAR2	AAGGGGAGGTCAGTTCATGTT	GCCTGGTCGGTTAGTTTTTGG
ncbi_104593066	Nnactin	GCGTTCTGCCGTCTTCTAAA	CCCTCTTGGATTGTGCCTC
	NnPTC1c<br>ds	CGGCATGACACAGTTGAACCTTAC<br>TGGG	GCCGCTTATGGTAGGGAAGGAAGG<br>AGG
	NnPTC1<br>PIR-SalI:	GCAGAATCTGAATTCGTCGACATG<br>ACACAGTTGAACCTTACTGGG	GCTCGAGAAGCTTGTCGACATGGT<br>AGGGAAGGAAGGAGG
	NnPTC1-1<br>A-BamHI	CCAATTCAGTCGACTGGATCCATG<br>ACACAGTTGAACCTTACTGGG	CGAGTGCGGCCGCGAATTCATGGT<br>AGGGAAGGAAGGAGG
