# Supplementary figures and images for: Cytological and Molecular Mechanism of Low Pollen Grain Viability in a Germplasm Line of Double Lotus
Source: Plants (Basel). 2023 Jan 13;12(2):387. doi: 10.3390/plants12020387 (PMC9867118; doi:10.3390/plants12020387)

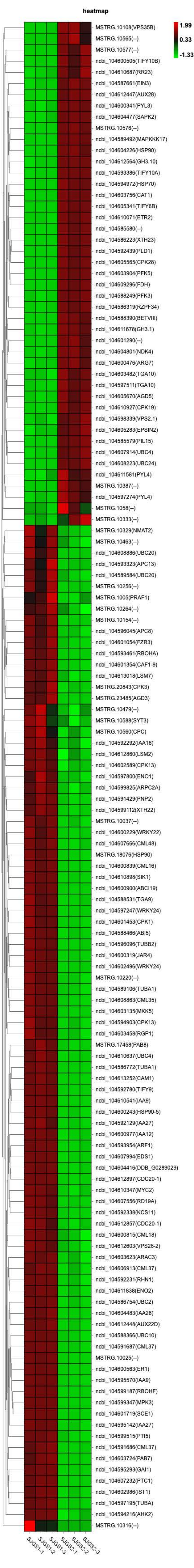

Supplement: Supplementary file 1 [file plants-12-00387-s001.zip › ╕╜╝╙╬─╝■/Figure S1 Heatmap of 135 differentially expressed genes.pdf]
